# No evidence of a causal relationship between hypothyroidism and glaucoma: A Danish nationwide register-based cohort study

**DOI:** 10.1371/journal.pone.0192311

**Published:** 2018-02-14

**Authors:** Marianne Thvilum, Frans Brandt, Thomas Heiberg Brix, Laszlo Hegedüs

**Affiliations:** 1 Department of Endocrinology and Metabolism, Odense University Hospital, Odense C, Denmark; 2 Department of Internal Medicine, Hospital of Southern Denmark, Sonderborg, Denmark; Universidad Miguel Hernandez de Elche, SPAIN

## Abstract

**Background:**

An interrelationship between hypothyroidism and glaucoma, due to a shared autoimmune background or based on deposition of mucopolysaccharides in the trabecular meshwork in the eye, has been suggested but is at present unsubstantiated. Therefore, our objective was to investigate, at a nationwide and population-based level, whether there is such an association.

**Subjects and methods:**

Observational cohort study using record-linkage data from nationwide Danish health registers. 121,799 individuals diagnosed with a first episode of hypothyroidism were identified and were matched with 4 non-hypothyroid controls according to age and sex. Prevalence of glaucoma was recorded and cases and controls were followed over a mean of 7.1 years (range 0–17). Logistic and Cox regression models were used to assess the risk of glaucoma before and after the diagnosis of hypothyroidism, respectively.

**Results:**

Overall, we found a higher prevalence of glaucoma in subjects with hypothyroidism as compared to controls (4.6% vs. 4.3%, p < 0.001). Prior to the diagnosis of hypothyroidism, the odds ratio (OR) was significantly increased for glaucoma [1.09; 95% confidence interval (CI): 1.04–1.13]. Based on the Cox regression model, there was no increased risk of glaucoma after the diagnosis of hypothyroidism [hazard ratio (HR) 1.00; 95% CI: 0.96–1.06], and the HR decreased further after adjusting for pre-existing co-morbidity (0.88; 95% CI: 0.84–0.93).

**Conclusions:**

There was an increased risk of glaucoma before but not after the diagnosis of hypothyroidism, suggesting that screening for glaucoma in hypothyroid individuals is unwarranted.

## Introduction

Hypothyroidism is a common endocrine condition, which has a female predominance and occurs in approximately 1–2% of the general population [1]. The most common causes of hypothyroidism are autoimmune thyroiditis or the consequence of previous surgery or radioiodine treatment[1, 2]. Irrespective of its cause, the standard treatment is levothyroxine[3]. However, and despite treatment, hypothyroidism has been associated with excess somatic[4] and psychiatric morbidity[5] as well as increased mortality[6]. Despite the very prevalent eye manifestations and accumulation of hydrophilic hyaluronan in the orbital space in Graves’ disease[7], we could, in a recent study, not demonstrate a significant association between hyperthyroidism without Graves’ orbitopathy and glaucoma[8]. However, whether hypothyroidism is associated with increased intraocular pressure is still debated[9].

Worldwide, glaucoma is among the leading causes of vision impairment [10], and the pathological mechanism, a dysfunction of the trabecular meshwork, is well described[11]. Furthermore, it has been hypothesized that hypothyroidism is associated with a deposition of mucopolysaccharides in the trabecular meshwork which initiates decreased aqueous outflow in addition to increased intraocular pressure leading to glaucoma[12]. This hypothesis has been supported by the finding of a lowering of the intraocular pressure after restoration of euthyroidism following thyroid hormone therapy[13]. In addition, an interrelationship between hypothyroidism and glaucoma due to a shared autoimmune background has also been suggested but is at present unclarified[14, 15]. However, as pointed out in our recent systematic review[9], the interpretation of the studies dealing with the relationship between hypothyroidism and glaucoma is hampered by methodological shortcomings such as major differences in the sample size, definition of disease phenotypes, lack of appropriate control for co-morbidity and, importantly, focus on the direction of such a potential association.

The aim of the present study was to investigate, at a nationwide level, whether there is an association between hypothyroidism and glaucoma, and if so to determine its direction. Denmark holds a long tradition for collecting data into well-established nationwide health and administrative registers[16]. By virtue, they provide an excellent resource for epidemiologic research that allows longitudinal follow-up of individuals without any significant selection or loss to follow-up. From the complete registration of admissions to hospitals, outpatient treatments and treatments in general practice as well as in eye clinics, hypothyroid individuals and patients treated for glaucoma can be ascertained with high accuracy. As another advantage, co-morbidity can be controlled for to a higher degree than hitherto.

## Materials and methods

### Data sources

We utilized The Danish Civil Registration System (DCRS), The Danish Demographic Database (DDB), The Danish National Patient Registry (DNPR) and The Danish National Prescription Registry (DNPrR), which are nationwide registers covering information such as demographics, date of death, hospital treatments and prescriptions of drugs on all persons living or having lived in Denmark from 1968[16]. All databases are hosted at Statistics Denmark[16] and have been described in detail[6]. The CPR-number, a unique 10-digit personal identification number assigned to all persons living in Denmark, allows record-linkage between all the databases on an individual level.

The present project is approved by the Danish Data Protection Agency and by Statistics Denmark to access health records.

### Hypothyroidism

Information on thyroid status was drawn from DNPR and DNPrR. Hypothyroidism was defined by the ICD-10 codes E03.2-E03.9, as recorded in the DNPR, or by at least two dispensed prescriptions of thyroid hormone (ATC = H03A), as recorded in the DNPrR. Subjects diagnosed after the age of 18 with primary hypothyroidism were eligible for the present study. As a consequence, we excluded individuals diagnosed with malignant thyroid diseases, congenital hypothyroidism or pituitary hypothyroidism, represented by the ICD-10 codes C73, E00.0-E00.9, E03.0-E03.1, E22.0-E22.9, E23.0-E23.7 and E24.0. Furthermore, cases diagnosed with hyperthyroidism (defined by the ICD-10: E05.0 to E05.9 in DNPR or having received dispensed prescriptions of antithyroid drugs, ATC = H03B in DNPrR), prior to the diagnosis of hypothyroidism, were excluded.

### Glaucoma

As for hypothyroidism, the information on glaucoma was drawn from DNPR and DNPrR. In DNPR glaucoma was defined by the ICD-10 codes H4.00-H4.09, H4.20 and H4.28. In DNPrR glaucoma was defined by at least two dispensed prescriptions of antiglaucoma medication (ATC = S01E).

### Study sample

The study sample was based on the entire Danish population identified from DCRS[16]. In order to investigate incident cases—and to obtain the same time frame of observation in DCRS, DNPR, and DNPrR—only individuals diagnosed with hypothyroidism after December 31, 1995 were included. In all, 121,799 individuals with a diagnosis of hypothyroidism were matched 1:4 according to age and gender with 487,196 non-hypothyroid controls. All participants were followed until migration, death, or December 31, 2013, whichever came first.

### Co-morbidity

The burden of co-morbidity was evaluated by using the Charlson score (CS). CS accounts for 19 disease groups (myocardial infarction, heart failure, vascular disease, cerebrovascular disease, dementia, chronic lung disease, rheumatic disease, gastric ulcer, liver disease, diabetes mellitus without complications, diabetes mellitus with complications, hemiplegia, kidney disease, cancer, cancer with metastases, lymphoma, leukemia, liver failure, and AIDS) by creating a weighted score on an individual level, to optimize the prediction of the one-year mortality risk[17]. The CS has been validated and used in different phenotypes, including non-malignant diseases[18].

The CS prior to the diagnosis of hypothyroidism, was used in the adjusted analyses of the risk of glaucoma following the diagnosis of hypothyroidism. For subjects with hypothyroidism, the CS reflects the time period from January 1, 1995 until the index-date (date of the diagnosis of hypothyroidism). In controls, the CS covers the time period from January 1, 1995 until the index-date of the corresponding case.

### Data analyses

For descriptive analyses, group frequencies were compared with the Pearson X^2^ test, whereas group means and medians were compared by a t-test and Mann-Whitney U test, respectively. In case of paired comparisons, the paired t-test was used.

The risk of glaucoma prior to the diagnosis of hypothyroidism was evaluated in a logistic regression analysis adjusted for age and sex. Following the diagnosis of hypothyroidism, the relationship between hypothyroidism and glaucoma was evaluated by a Cox regression model. Age was chosen as the underlying time variable. In both cases and controls, person years of follow-up were accumulated from the index-date of the case and terminated on the date of diagnosis of glaucoma, migration, death, or end of follow-up (December 31, 2013), whichever came first.

In all Cox analyses the variable “pair” (equals 1 case and 4 controls) was used as a stratum variable, fixing the baseline hazard within a matched pair, while at the same time allowing this baseline hazard to vary freely between pairs. Subsequently, all Cox regression analyses were adjusted for the degree of co-morbidity preceding the diagnosis of hypothyroidism. The data was stratified for the register they were identified from, either DNPR or DNPrR, in order to evaluate potential differences.

As hypothyroidism is associated with increased mortality[6], cases *á priori* have a shorter observation period than the controls and, at least theoretically, a lower risk of being diagnosed with glaucoma. To account for a potential influence of competing risk, the risk of glaucoma was re-evaluated by the method of Fine and Gray[19].

Significant differences were defined as a p-value below 0.05, using two-tailed tests. All analyses were conducted using STATA version 13.0 (2013; Stata Corporation, College Station, TX, USA).

## Results

### Baseline characteristics of the study sample

Characteristics of the cases and the controls from the background population are presented in Table 1. In all, 121,799 individuals fulfilled the criteria for hypothyroidism. This corresponds to a prevalence of 1.27%. The majority of cases (80,309) were identified from DNPrR solely, whereas about 1/3 of individuals (36,165) were registered in both DNPR and DNPrR. The mean follow-up time was 6.9 years for cases (range 0–17) and 7.2 years for controls (range 0–17). Controls had significantly lower burden of co-morbidity than the subjects with hypothyroidism (percent of controls and cases with CS = 0: 30% and 14%, respectively, p < .001). Also, controls had a significantly lower prevalence of glaucoma as compared to cases, 4.3% and 4.6%, respectively (p < .001).

**Table 1:** Baseline characteristics of hypothyroid cases and controls.

	Cases	Controls
**Number**	121,799	487,196
**Females, %**	81.7	81.7^3^
**Mean age^1^ (range)**	56.7 yr (18–104)	56.7^3^ yr (18–104)
**Mean follow-up time (range)**	6.9 yr (0–17)	7.2 yr (0–17)
**CS^2^ = 0, %**	14	30
**Identified from DNPrR solely**	80,309	-
**Prevalence of glaucoma, %**	4.6^4^	4.3^4^

1: Mean age at the time of diagnosis

2: Pre-existing co-morbidity as defined by the Charlson score

3: Controls were matched with cases according to age and gender

4: Significantly different

### The risk of glaucoma before the diagnosis of hypothyroidism

The results are shown in Table 2. Based on the logistic regression analysis, individuals with hypothyroidism had a significantly increased risk of having glaucoma prior to the thyroid diagnosis (OR 1.09, 95% CI: 1.04–1.13). Evaluating the risk of glaucoma before the thyroid diagnosis, but censoring diagnoses made within 365 days prior to the diagnosis of hypothyroidism in order to evaluate potential confounding by indication (Berkson’s bias) [20], yielded essentially similar results (OR 1.08; 95% CI: 1.03–1.13).

**Table 2:** Odds ratio for glaucoma prior to hypothyroidism.

	OR^1^
**All**	1.09 (1.04–1.13)
**Females**	1.09 (1.05–1.15)
**Males**	1.04 (0.95–1.13)
**DNPR^2^**	1.15 (1.08–1.22)
**DNPrR^3^**	1.05 (1.01–1.10)

1: Odds ratios, with 95% confidence intervals (CI)

2: Individuals identified from the Danish National Patient Registry

3: Individuals identified from the Danish National Prescription Registry

When stratifying for gender, we found a significantly increased risk of glaucoma before the diagnosis of hypothyroidism among females (OR 1.09; 95% CI: 1.05–1.15), but not among males (OR 1.04; 95% CI: 0.95–1.13). Stratification for type of register did not change the results significantly (Table 2).

### The risk of glaucoma after the diagnosis of hypothyroidism

There was a slight, albeit non-significant, excess risk of glaucoma in hypothyroid compared with control individuals [hazard ratio (HR) 1.05; 95% CI: 1.00–1.10], (Table 3). Albeit the result reached significance, it did not change significantly from the non-adjusted findings, when applying censoring of diagnoses made within 365 days following the diagnosis of hypothyroidism (HR 1.06; 95% CI: 1.05–1.07). Stratification for gender did not significantly influence this finding. Based on the competing risk analysis, according to the method of Fine and Gray[19], we found no increased risk of glaucoma after the diagnosis of hypothyroidism (HR 1.00; 95% CI: 0.96–1.06). In the analysis adjusted for co-morbidity, there was a decreased risk of glaucoma after the diagnosis of hypothyroidism, (HR 0.88; 95% CI: 0.84–0.93), (Table 2). Restricting the analysis to individuals without pre-existing co-morbidity (CS = 0), the HR decreased further (HR 0.67; 95% CI: 0.56–0.79). Again, stratification for type of register did not change the results significantly (Table 3).

**Table 3:** The risk of glaucoma following the diagnosis of hypothyroidism.

	HR^1^	HR, adjusted^2^
**All**	1.05 (1.00–1.10)	0.88 (0.84–0.93)
**Females**	1.05 (0.99–1.10)	0.90 (0.85–0.95)
**Males**	1.06 (0.95–1.19)	0.83 (0.73–0.94)
**CS^3^ = 0**	0.67 (0.56–0.79)	
**DNPR^4^**	1.06 (0.99–1.14)	0.93 (0.86–1.00)
**DNPrR^5^**	1.05 (0.99–1.11)	0.84 (0.79–0.90)

1: Hazard ratio, with 95% confidence intervals (CI)

2: Adjusted for pre-existing co-morbidity using the Charlson score

3: The Charlson score

4: Individuals identified from the Danish National Patient Registry

5: Individuals identified from the Danish National Prescription Registry

## Discussion

This is a population-based analysis of the interrelationship between hypothyroidism and glaucoma in a Danish nationwide study. Having access to more than 120.000 hypothyroid individuals, each matched with four controls, we found a positive association between hypothyroidism and glaucoma. As Cox power calculations estimate a necessary sample size of 3140 subjects, to show an increase in HR of 10% with 90% power, the sample size in the present study was sufficient to detect even relatively small differences between cases and controls.

Dividing the analyses into the period prior to and following the diagnosis of hypothyroidism, a significantly increased risk of glaucoma was seen before the thyroid diagnosis. Following the diagnosis of hypothyroidism, there was no increased risk of being diagnosed with glaucoma.

As shown in our recent meta-analysis, the literature suggests an increased risk of hypothyroidism following a diagnosis of glaucoma, and *vice versa*[9]. However, drawbacks of the previous studies, which evaluate the association between hypothyroidism and glaucoma, are: limited sample sizes, ambiguous definitions of hypothyroidism as well as glaucoma, short if any follow-up periods, and cross sectional designs, none of which favour robust conclusions with respect to causality[9]. Interestingly, the majority of the previous studies are in favour of an association between hypothyroidism and glaucoma. In contrast, a large longitudinal study from Sweden, including a register-based study sample, found no significantly increased risk of glaucoma following hypothyroidism[21]. However, definition of both hypothyroidism and glaucoma was based solely on the reported use of medication without any information regarding underlying reason for use of medication, such as diagnosis codes, nor was there adjustment for co-morbidities[21]. In the present study, we used a validated definition of hypothyroidism by utilizing the Danish nationwide registers[6]. As for glaucoma, the diagnoses were performed by ophthalmologists. Thus, including both data from the DNPR, representing individuals treated in a hospital setting in addition to DNPrR representing individuals from primary care, gives a very broad and unselected study sample, which minimizes selection and/or information bias[22]. Although information bias is a limitation in register-based studies, the validity of the DNPR is high and misclassification of thyroid dysfunction has been shown to occur in less than 2% of cases. Moreover, all diagnoses of glaucoma as well as all prescriptions of antiglaucoma medication were performed by ophthalmologists which lowers the risk of selection bias further [23]. Studied in this way, we found no increased risk of glaucoma after the diagnosis of hypothyroidism. This finding persisted after taking competing risk into account.

It is well-known that hypothyroidism is associated with a wide range of co-morbidities including cardiovascular diseases such as hypertension and diabetes [24, 25]. Since these conditions have also been found significantly associated with glaucoma [26, 27], failing to control for such important confounders most likely overestimates the association between hypothyroidism and glaucoma. Ours is the first study to utilize the validated CS in the analyses, and thereby covers a wide range of cardiovascular and autoimmune diseases in order to eliminate, or at least reduce, prominent confounders. When this is said, we did not apply directed acyclic graphs (DAGs) in our identification of confounders in the present study. The use of DAGs increases the possibility of empirically estimating causal association [28]. In line with this, we cannot know whether the co-morbidities may act as confounders or mediators, which should be noticed, when interpreting the results. However, as no *a priori* criterion developed for a specific application is guaranteed to be appropriate for confounder identification in general, this remains speculative [28]. In the analyses adjusting for morbidity, we found a decreased risk of glaucoma following the diagnosis of hypothyroidism. It seems counterintuitive that hypothyroidism *per se* should reduce the risk of developing glaucoma, as the results indicate. On the other hand, since we presume that all patients diagnosed and registered with hypothyroidism are treated for their thyroid disease, it could be hypothesized that the treatment protects against an increased pressure in the trabecular meshwork of the eyes, and thereby prevents glaucoma. In support, improvement of the intraocular pressure following thyroid hormone therapy and obtaining euthyroidism has been demonstrated[12, 13]. Not having access to patient records or longitudinal biochemical data in our large national cohort, this line of thought cannot be verified in our study and remains speculative.

Importantly, our findings should be interpreted in the light of potential limitations. First of all, we lack information on biochemical data and effect of treatment. While all included patients received treatment, based on prescription data according to the DNPrR, this does not include information on the effect of treatment or the severity of the disease. As a surrogate measure, we stratified the analyses into hypothyroid individuals identified from a hospital setting and from general practice, which represents the most affected and the least affected individuals, respectively. Based on these analyses, we found no indication of a dose-response (severity) relationship between hypothyroidism and risk of developing glaucoma. This would argue against a causal relationship[29]. Although we lack data regarding the etiology of hypothyroidism, it has previously been shown that hypothyroidism due to autoimmune thyroiditis accounts for more than 80% of all hypothyroid cases[1]. This is important to acknowledge, as also glaucoma etiologically includes aspects of autoimmunity[14, 15]. However, despite the fact that autoimmune diseases tend to co-exist[25] our data cannot support a significant association between autoimmune thyroiditis and glaucoma. In addition, we lack data concerning racial distribution among cases and controls. As glaucoma and to some degree also hypothyroidism has racial disparities with regard to diagnosis and progression, this is a limitation as well [30]. However, based on data from Statistics Denmark (www.dst.dk) we can assume that around 90% of the study sample, as indeed of the general population in Denmark, is Caucasian. It could also be speculated that the follow-up period, which although 7 years, is not long enough. As for strengths, we were able to stratify for the period before and after the diagnosis of hypothyroidism, which contrasts the majority of previous studies, mainly characterized by cross sectional designs[9]. This offers an important temporal perspective and thereby novelty. What remains is a discretely increased risk of glaucoma patients subsequently being diagnosed with hypothyroidism.

In conclusion, we found a minute increased risk of glaucoma before but not after the diagnosis of hypothyroidism, whether this is an effect of the treatment of hypothyroidism needs further study.
